# Rarely Recognized Antibody Diversification in Covid-19 Evolution to Counteract Advanced SARS-CoV-2 Evasion Strategies, and Implications for Prophylactic Treatment

**DOI:** 10.3389/fphys.2021.624675

**Published:** 2021-08-03

**Authors:** Siguna Mueller

**Affiliations:** Independent Researcher, Kaernten, Austria

**Keywords:** antibody diversification, immunogenic peptides, amino-acid sequence dependency, epitope vaccines, post-translational modification, vaccine design, Covid-19, rarely recognized immune responses

## Abstract

The ongoing Covid-19 pandemic underscores the importance of finding effective and safe ways to combat the virus, and to optimally understand the immune response elicited upon natural infection. This likely involves all components of the immune system, both innate and adaptive. The impetus for the rapid development of prophylactic treatment options has led to an intense focus on neutralizing antibodies (Abs), and many novel and specialized platforms have been designed to achieve that goal. B-cell immunity relies on the generation of a diverse repertoire of Abs. Their structural variation is defined in terms of amino acid composition that is encoded in the genome or acquired through somatic mutations. Yet, key examples of frequently neglected antibody diversification mechanisms involving post-translational modifications such as N- or O-linked glycosylation are present in significant portions of the population. During the last few years, these and other beyond gene sequence determined humoral immune response mechanisms have in some specific cases revealed their potent immunomodulatory effects. Nonetheless, such more unusual mechanisms have not received much attention in the context of SARS-CoV-2. Thus, with specific focus on the latter, this paper presents, (1) the rationale for considering beyond sequence determined strategies, (2) evidence for their possible involvement in Covid-19 disease evolution, (3) consequences for vaccine design exemplified by one of the vaccine candidates that is currently undergoing trial, and (4) more general implications. Based on a critical interpretation of published literature, the hypotheses developed in this study point to a crucial role of non-genetic antibody diversification mechanisms in disease evolution to counteract unique immunogenicity determinants of SARS-CoV-2 infection. The involvement of post translational mechanisms may also help explain the widely varied immune response observed, not only among different patient groups, but also in terms of their observed incompatibility with SARS-CoV-2 infection in several human cell types. The article highlights potentials and challenges of these refined humoral immune response mechanisms to most optimally target non-genetic viral evasion strategies.

## 1. Motivation

The ongoing Covid-19 pandemic has triggered intense global R&D activity to develop safe and effective prophylactic and therapeutic options against the disease. The scale of the humanitarian and economic impact of the COVID-19 pandemic has been driving the need to exploit next generation-approaches for increased speed and manufacture, and is reflected in the vaccines to first obtain regulatory approval, as well as many of the second-generation alternatives still in development.

The main focus of most of the vaccine approaches against SARS-CoV-2 builds on the discovery and development of specific antibodies (Abs) to achieve neutralization. With such efforts, the primary target is the viral spike that is believed to be responsible for binding to the ACE2 receptor on the host cell (Hoffmann et al., 2020; Walls et al., 2020; Wang Q. et al., 2020; Zhou et al., 2020).

While the isolation and characterization of specific Abs that target epitopes on the viral spike (Liu et al., 2020; Walls et al., 2020) has revealed specific regions that seem to be immunogenic, some of the challenges with such methods are, (1) most potently neutralizing antibodies—as have been generated from convalescent individuals—seem to only contribute little to the overall neutralizing antibody response (Weisblum et al., 2020); (2) analysis of convalescent plasma samples that are potentially neutralizing do not always have corresponding Abs (Weisblum et al., 2020); (3) although the innate immune system is crucial for Covid-19 disease clearance, its interplay with the acquired immune system is not adequately understood; (4) there is a huge diversity of neutralizing antibody responses within and between individuals (Weisblum et al., 2020). In the context of treatment with monoclonal Abs (mAbs), which are obtained from a convalescent patient's plasma and generated by cloning a unique white blood cell, obtaining a complete selection of safe and neutralizing mAbs for an entire population is very challenging. This is even more difficult as the individual immune response of Covid-19 patients is known to depend on co-morbidities and existing pathologies (Dorward et al., 2020); (5) SARS-CoV-2 infection and replication capacity varies drastically between different cell lines and types (Harcourt et al., 2020); (6) focusing on the spike protein only (or on additional viral targets) allows only for the selection of a fixed set of epitopes and their corresponding antibodies and cannot counteract ongoing viral evolution.

Overall, identifying the complete landscape of the diversity of the SARS-CoV-2 neutralizing antibody responses is particularly challenging. Numerous factors, including those based on inadequate immune responses or suboptimal monoclonal antibody treatment might drive the selection of mutated viruses that have acquired resistance to commonly occurring antibodies (Baum et al., 2020; Weisblum et al., 2020). Moreover, some viral mutates detected in a Europe strain seem to bind to T cell receptors with a significant increase in binding affinity (Cheng et al., 2020).

Sørensen et al. (2020) suggest a radically different approach for SARS-CoV-2 vaccine development that merits consideration (see **Figure 2** below for a summary of their approach, and Table 1 for its promised benefits). Rather than focus on a set of receptor-binding interference with the ACE2 receptor alone, these authors differentiate the viruses' mechanism of action and its increased pathogenicity. Given that we have been exposed to previous coronaviruses without serious global disasters, this approach of trying to identify the singularity of SARS-CoV-2 as the driver of our current pandemic should be seen as a plausible and valid alternative.

**Table 1:** Design features of Biovacc-19 aimed to minimize and prevent known risks.

**Targeted difficulty or challenge**	**Design feature of Biovacc-19 that aims to address this (Sørensen et al., 2020)**
The development of antibody resistance in form of SARS-CoV-2 mutants that escape antibody neutralization.	
•It is difficult to predict the degree to, and pace at which SARS-CoV-2 might evolve to evade neutralizing antibodies. •Although this virus mutates less frequently than some other pathogens, some mutations have already led to variants of concern (Centers for Disease Control and Prevention, 2019; Callaway, 2020). Some of the mutations confer resistance to monoclonal antibodies or convalescent plasma (Weisblum et al., 2020) or enhance the superantigenic character of the spike glycoprotein (Cheng et al., 2020).	By assembling a set of different epitopes, it is expected that the recombinant protein of this peptide vaccine will exhibit a sufficient sequence variation to deny the virus the various cell receptor binding options. In addition, the design strategy includes the *a priori* use of a specific adjuvant to give the necessary enhancement in T-Helper 2 (TH-2) response to the peptide specific epitopes.
Autoimmune (AI) diseases triggered by vaccination. Reports on AI diseases following vaccination have accumulated in the last decade (Wraith et al., 2003; Vadalà et al., 2017; Segal and Shoenfeld, 2018), but the pathogenic mechanisms by which vaccines can cause AI reactions have not been fully elucidated (see also sections 3.5, 3.6).	The reliance on non-human like (NHL) epitopes is suggested to prevent local or systemic toxicity.
The concern of antibody-enhancement (ADE). One of the established safety concerns in vaccine development is the possibility of ADE. While amino acid variability and antigenic drift may be key contributors (Sørensen et al., 2020), the mechanisms causing ADE are still unclear (see also Sections 3.5 and 3.6).	Since the antibodies are directed toward the binding domain of both the main receptor and the suggested co-receptor domains, the danger of ADE is proposed to be low.

A logical and critical analysis of the rationale and method of action of Biovacc-19 raises several immediate questions. The vaccine peptides constituting Biovacc-19 are a combination of various sequences of five amino acids found on the Spike protein (see Box 1 for the rationale and method of action of Biovacc-19). These claims are based entirely on a rolling window search in terms of 6 amino acids (Sørensen et al., 2020, Table 1). This is concerning as antibodies can recognize 5–6 amino acids—as was also mentioned by Sørensen et al. (2020). Now, with SARS-CoV-2 this is particularly disturbing as one insert in alignment 6 in the spike protein has three positive Arginines in combination with a Proline. In Sørensen et al. (2020) this is recognized to be critical to secure membrane anchoring. Importantly, it was pointed out that this is “not acting in the same way as a typical cell penetration peptide due to there being only four amino acids.”

Box 1 **Biovacc-19:**

The question regarding the involvement of beyond sequence determined adaptive immune responses in Covid-19 arose when analyzing one of the candidate vaccines that is in advanced pre-clinical development (Sørensen et al., 2020). The rationale and method of action of Biovacc-19 is summarized in **Figure 2**. Biovacc-19 is a peptide vaccine. The individual peptides have been selected by blasting the spike protein sequence using moving window of 6 amino acids against the human protein sequence database on Uniprot (Uniprot–P0DTC2) (Sørensen et al., 2020). Consequently, a combination of various amino acids found on the viral Spike protein are placed in scaffolds and deployed as antigens. Because of this combination, it is argued that it will be as if a medium sized protein was used as vaccine antigen “resulting in large sequences variation and hence giving a surplus of Th2 epitopes” (Sørensen et al., 2020).

The developers posit that Biovacc-19 will not only be effective. Its unique logic of design is expected to eliminate the risk of creating an ineffective or actively harmful vaccine, especially related to antibody dependent enhancement (ADE) or autoimmune (AI) conditions (for details, see Table 1). In particular, it is argued that since Biovacc-19 is composed exclusively of non-human-like (NHL) sequences, that the likelihood for getting local or systemic toxicity is minimized.

A second question arises relative to additional mechanisms that the virus could use beyond mere sequence-based interactions, and how our immune system may be responding to these. In fact, the very findings in Sørensen et al. (2020) point to the involvement of advanced viral tricks, including the above-mentioned binding mechanism involving <6 amino acids, and special strategies that utilize charge, salt bridges, and conformation. Indeed, the key differences described in Sørensen et al. (2020) between SARS-CoV and SARS-CoV-2 are based on strategies far beyond the interaction between the S protein and its receptor ACE2 that most other platforms focus on.

In spite of the numerous R&D activities in place, there has not been much effort to assess the role and impact of immune responses more complex than to short specified amino acid sequences induced by SARS-CoV-2 infection. Some pre-corona studies (and related to other viruses) (Van De Bovenkamp et al., 2018) have revealed that these strategies may have both positive and negative effects on antibody affinity and that they exhibit distinct patterns according to certain (patho)physiological conditions of the host.

**Figure 1:**
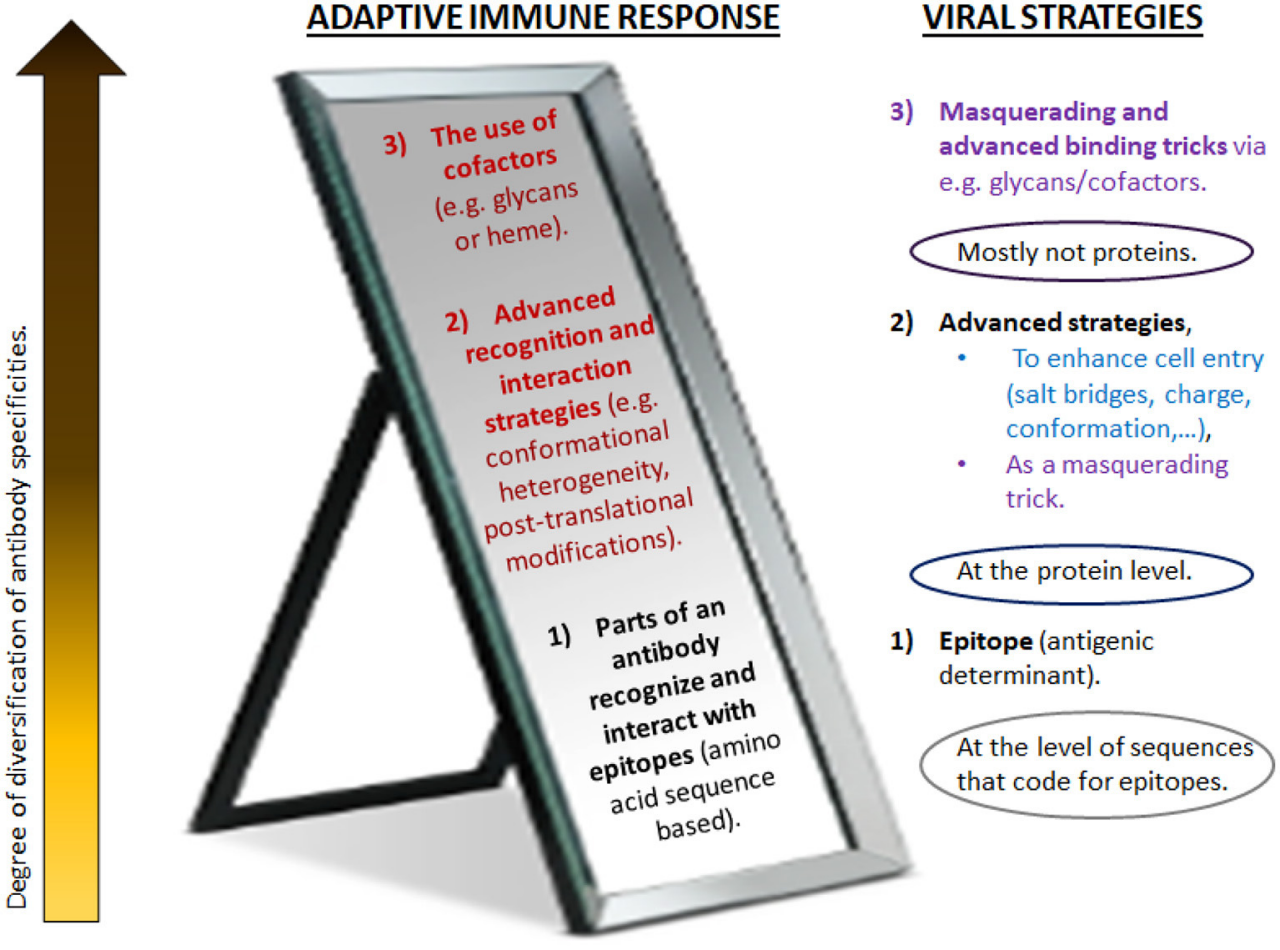
Different levels of antibody diversification counteract (or mirror) various masquerading tricks of pathogens. Traditionally, adaptive immunity has focused on genetic mechanisms that give rise to the diversity of antibody V regions. These take place during B cell development in the bone marrow and upon encounter of antigen in the periphery. For increased specificity, refined receptors are acquired through somatic mutations and irreversible genetic recombination of antigen receptor segments. In contrast to more traditional vaccines, synthetic vaccines utilize short amino acid sequences of the immunogenic protein (epitopes) to evoke the anticipated antibody response. As this approach distinctively relies on the specific amino acid sequence of the immunogenic protein of interest, this is believed to induce a direct and potent immune response. However, the immune system has a much greater level of adaptability and plasticity than usually considered (here, depicted as higher levels). It mirrors various advanced masquerading tricks of pathogens (right) and goes far beyond what is encoded in the genome or generated by the acquisition of somatic mutations. Likely, the immune response is highly variable and interlinked, in order to simultaneously employ the most optimal strategies to combat the viral strategies (**Figure 4**). Advanced strategies beyond the main receptor binding paradigm as suggested in Sørensen et al. (2020) for SARS-CoV-2 are depicted in blue; the strategies employed for coronaviruses in general are in purple. Red/dark red: frequently neglected strategies for remodeling V regions, as suggested to be a key factor in Covid-19. Many of these seem to be influenced by metabolism and the pathophysiologic state of host. (For details, see text).

This paper posits that these are key factors in a healthy Covid-19 disease response, or, if misdirected, drivers of pathogenicity and mortality. Nonetheless, here it is not merely about one single hypothesis such as a certain relationship based on a very specific involvement of these mechanisms, but to further the development of a broader theory regarding the reflective nature of the adaptive immune system and non-genetic viral immune evasion strategies. It will be argued that these processes mirror each other on many levels. Both the virus and the immune system are equipped with strategies ranging from mere nucleotide-based determinants to more plastic and flexible refinement mechanisms (Figure 1).

The central question to be addressed in this paper, with special focus on Covid-19, is then: is there a - potentially critical - role of an adaptive immune response beyond what is encoded in the genome or generated by the acquisition of somatic mutations? Section 2 will gather evidence for the possible involvement of such alternative immune response mechanisms in disease evolution; implications for vaccine design will be evaluated in section 3, using Biovacc-19 as a case analysis; section 4 offers some discussion, and conclusions are given in section 5.

## 2. The Possible Role of Rarely Recognized Antibody Diversification in Covid-19

Recent studies related to Covid-19 disease pathology have revealed some paradoxical findings. For example, convalescent individuals often exhibit low levels of plasma neutralizing activity, even with patients from whom potent neutralizing antibodies have been isolated (Robbiani et al., 2020; Wu F. et al., 2020). On the other hand, while specific and very potent antibodies were isolated from other convalescent plasma samples, it was found that these antibodies contributed little to the overall neutralization activity of plasma from the very same individual (Weisblum et al., 2020). These authors concluded that “the most potently neutralizing antibodies generated in a given Covid-19 convalescent individual may contribute in only a minor way to the overall neutralizing antibody response in that same individual.”

All this points to the urgent need to better understand the mechanisms underlying Covid-19 neutralization. Many questions remain unresolved. For example, Wang C. et al. (2020) identified a monoclonal antibody (47D11) that neutralizes “SARS-CoV and SARS-CoV-2 through a yet unknown mechanism that is different from receptor-binding interference.” Moreover, some studies (Grifoni et al., 2020; Moderbacher et al., 2020) that analyzed the immune response in acute and convalescent subjects revealed some interesting results. Individuals that were fully recovered had measurable antibody, helper and killer T cell responses, whereas in contrast, the adaptive immune response in acute Covid-19 patients varied widely. Finally, Wu Y. et al. (2020) suggest that some of the differences between SARS-CoV and SARS-CoV-2 Abs may be due to certain hydrophilic interactions at the interface between these antibodies and the receptor binding domain (RBD). The structural basis for one of these (B38—which blocks the interaction between the RBD and the ACE2 receptor) was linked to specific steric hindrances.

Overall, these findings suggest various refined immune strategies that heavily rely on factors other than the amino acid composition of the immunogetic peptide alone. Generally, it is accepted that the immune response to SARS-CoV-2 is multi-layered, involving both cell-mediated immunity and antibody production (Azkur et al., 2020; Vardhana and Wolchok, 2020). Nonetheless, the role of non-genetic mechanisms of antibody diversification has not received broad attention in the context of SARS-CoV-2. Inspired in large part by the review (Kanyavuz et al., 2019), this section analyzes the hypothesis that such unassessed mechanisms play a more important role in the immune response in Covid-19 than previously anticipated. In fact, it has only been during the last few years that the significance of alternative mechanisms has been recognized, as they vastly extend the traditional B-cell response paradigm (see Kanyavuz et al., 2019 for a review). This was made possible by new technologies, including high- throughput sequencing of the entire human B cell repertoire (Wardemann and Busse, 2017; Imkeller and Wardemann, 2018), and isolation and characterization of human monoclonal antibodies via advanced epitope mapping techniques (Corti and Lanzavecchia, 2013; Klein et al., 2013; Liu et al., 2020).

### 2.1. Multi-Tire Action–of Both the Virus and the Adaptive Immune Response

Strategies for modulating the functional activities of antibodies can significantly effect antigen binding (Van De Bovenkamp et al., 2018). Adaptive immunity responses defined in terms of amino acid composition can even be overruled by such additional mechanisms (Kanyavuz et al., 2019).

A key example of this is given by the glycosylation of antibody variable (V) regions. Glycoproteins produced by plasma cells are some of the most important components of B-cell immunity. The most important class of such immunoglobulins is IgG. Notably, however, while all IgGs contain glycans, the V domains of the antigen- binding fragment (Fab) arm may also acquire *N*- or *O*- linked glycans as a result of post-translational modifications. The emergence, function, and regulation of such IgG glycans has only recently been elucidated.

For instance, a recent study identified variable domain glycosylation as a significant modulator of antigen binding. Notably, when a panel of Fab-glycosylated human IgG monoclonal antibodies was mutated at the glycosylation sites back to the germline residues, it was found that in most IgGs the mutations abrogating the glycosylation of the V region resulted in a significant decrease in the binding affinity for the target antigen (Van De Bovenkamp et al., 2018). Another study (Jacquemin et al., 2006) investigated the human IgG alloantibody that neutralizes the procoagulant activity of factor VIII (FVIII) and revealed an interesting relationship between antibody binding and neutralization. It was shown that the functional activity of this antibody relies heavily on an unusual modification—again, the presence of a V-linked glycan. Surprisingly, it was found that while the oligosaccharides present in the variable part of the heavy chain of this anti-FVIII antibody did not affect its affinity for FVIII, it significantly increased the inhibition of FVIII activity, possibly by steric hindrance of its active site mediated by these glycans.

Glycosylation is a key example how antibody binding specificities and affinities can be extended in this two-tired process via those types of post-translational modifications. This process seems to mimic and counteract viral immune evasion strategies via the help of glycans, for coronaviruses in general, and SARS-CoV-2 in particular (see section 3.1).

It is important to note that Sørensen et al., 2020 identified a two-factor mode of action of SARS-CoV-2 in addition to binding to ACE2 receptors. These authors posit that this virus is able to bypass this pathway altogether and utilize an alternative co-receptor dependent phagocytic method of action. The latter is believed to be facilitated by cumulative charge from sections on the Spike surface placed at positions that enable efficient binding via salt bridge formations. These unique binding and cell entry strategies were identified in another study as well (Cheng et al., 2020) and represent other facets of a multi-tiered viral system.

These different viral strategies will likely be mirrored by the immune system in an analogs manner as with glycosylation. Thus, in terms of an adaptive immune response, it seems plausible that in addition to gene sequence determined response processes, the immune system will also try to mount non-genetic diversification mechanisms, to oppose the various strategies the virus is using (Figure 1).

**Figure 2:**
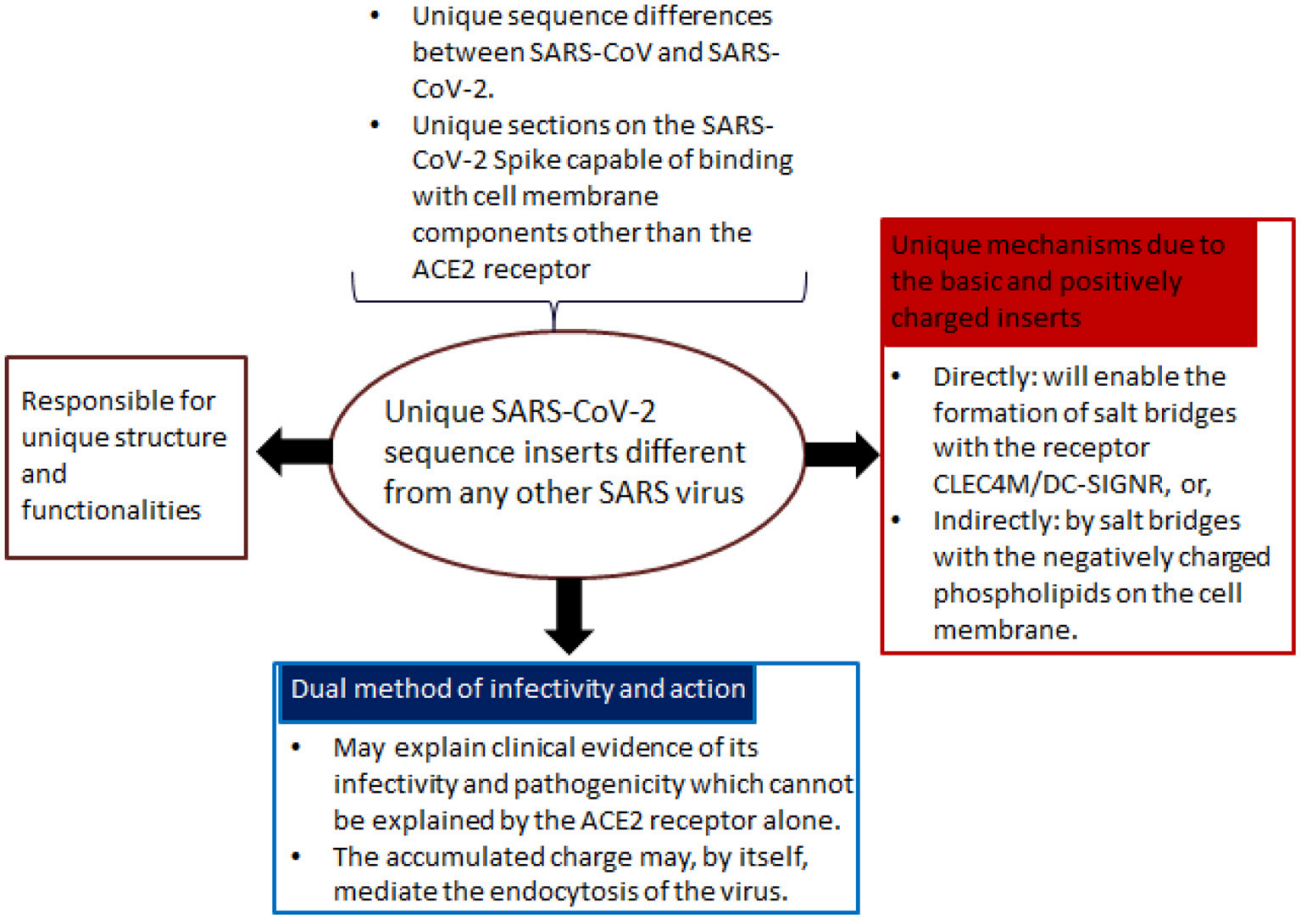
Rationale for the development of Biovacc-19. In Sørensen et al. (2020), the developers proposed a radically new approach for vaccine design: firstly, they identified unique sequence inserts into the virus, different from other SARS viruses; secondly, based on these unique inserts, a singular mode of action is identified, both in terms of its infectivity and pathogenicity. Based on their analysis, SARS-CoV-2 can enter cells without using the ACE2 main receptor. This is important for treatment and vaccine development strategies as the binding with cell membrane components is strongly facilitated by the unique molecular structure of the positively charged Spike trimer surface.

**Figure 3:**
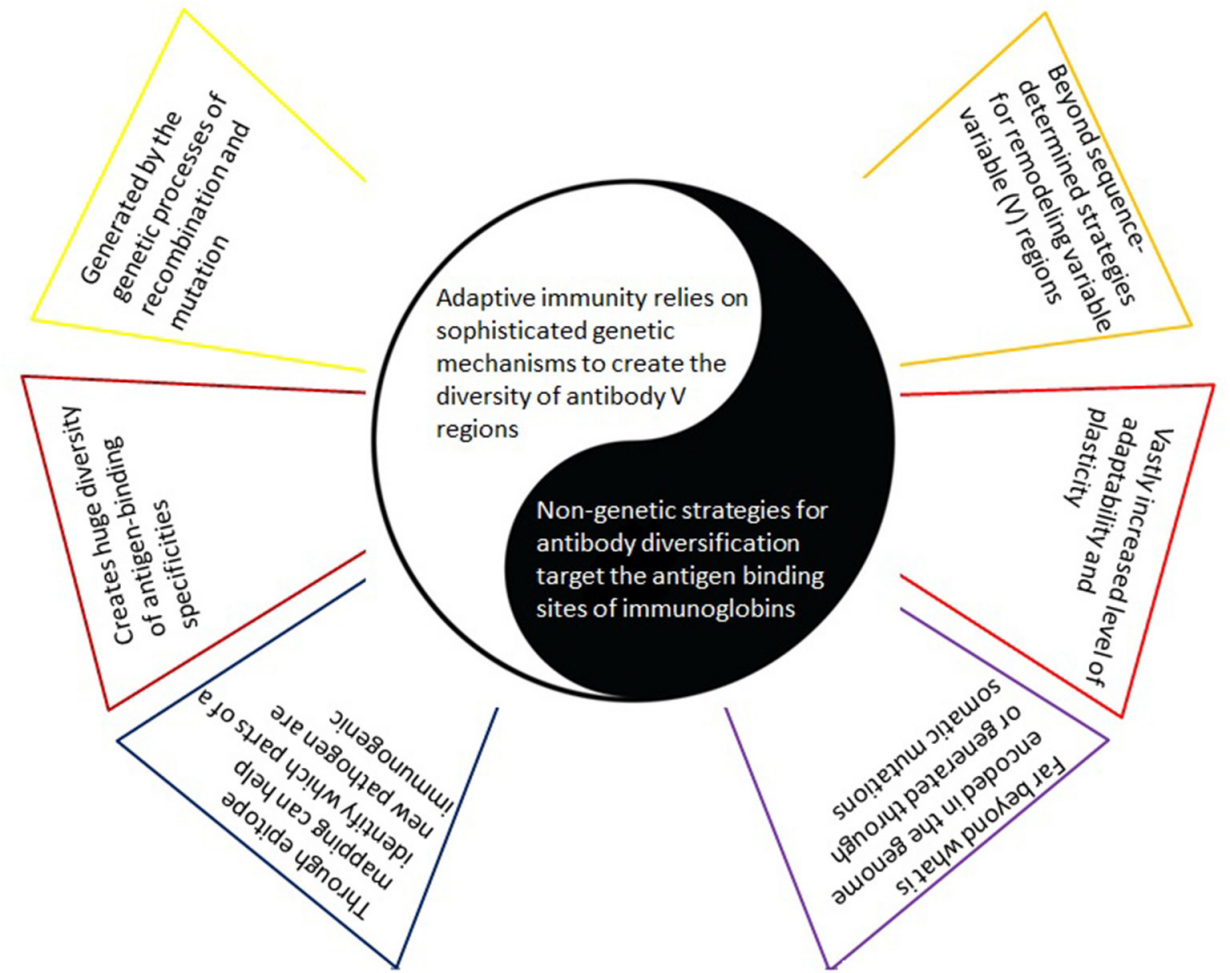
Adaptive immunity based on the usually considered paradigm versus alternative antigen-binding specificities via non-genetic strategies. With the former (left), there is an implicit understanding that a predetermined set of amino acid sequences - representing one or more epitopes on the synthesized vaccine - will trigger the generation of the corresponding antibodies - as a clear and direct response to these amino acid sequences only. In contrast, complementing (yet in many ways opposing) non-genetic mechanisms for improved antigen-binding specificities (right) lead to a much greater level of adaptability and plasticity and include the insertion of non-immunoglobulin proteins, post-translational modifications, the use of non-protein cofactor molecules for antigen recognition, and conformational heterogeneity of the antigen binding site.

Notably, the co-receptor dependent mode of action identified in Sørensen et al. (2020) heavily relies on charge, steric influences, and conformation (Figure 2). And indeed, each of these latter factors are also key components of antibody refinement responses that utilize more than mere gene-based determinants (Figure 3). Thus, to combat SARS-CoV-2, a successful neutralization response will likely involve a multi-tiered approach of the immune system in order to mirror the corresponding viral strategies.

A loss of clonal diversity and a contraction of naive T cells with proliferative capacity has been suggested to be associated with age-related waning of adaptive immune function and may in part help explain the association of Covid-19 disease severity with age (Vardhana and Wolchok, 2020). Analogously, it seems possible that the plasticity of these adaptive responses weakens with age in an analogs manner of a B cell related immunosenescence. More generally, severe morbidity and death may arise in situations of a limited or impaired spectrum of non-genetic immune responses, as dictated by unique pathophysiological factors of the host (see section 3.2).

### 2.2. Immune Responses More Complex Than to Short Specified Amino Acid Sequences May Be Necessary to Combat Viral Co-receptor Strategies

It is possible that SARS-CoV-2 uses, in addition to ACE2, additional receptors for attachment (Sørensen et al., 2020). If so, usually considered antibody diversification may not be able to adequately neutralize the virus. On the other hand, more unusual immune response mechanisms are often sufficient to neutralize those viruses that employ co-receptor strategies for infection (Kanyavuz et al., 2019). In particular, one study analyzed patient-derived anti-HIV-1 antibodies endowed with special post-translational strategies. It was found that certain HIV-1 neutralizing antibodies have extra sulfate groups added to Tyrosine residues in their complementary-determining region. This study showed that the binding site for a particular HIV-1 co-receptor closely overlaps with the epitopes of the Tyrosine sulfated antibodies. In fact, these co-receptor components and the corresponding sulfated antibody adopt an almost identical configuration while binding to the same binding site on the HIV-1 gp120 envelope glycoprotein (Huang et al., 2007). As a result, the presence of sulfotyrosines in these antibodies leads to some molecular mimicry and competition: antibody binding of the virus is analogs to the binding of the co-receptor molecule to the virus. It is found that this antibody sulfation was critical for virus neutralization, and antibody-expressing cells lacking this post-translational modification lost this particular neutralization ability.

As SARS-CoV-2 seems to be able to use mechanisms in addition to main receptor binding (Sørensen et al., 2020), one can suggest that even if binding to ACE2 was prevented, that the virus would still be able to attach to its coreceptor. In that case, a more refined strategy similar to the above may be necessary to neutralize SARS-CoV-2 infection.

If the immune system was able to deploy strategies such as Tyrosine sulfation or other diversification strategies to extend its antibody repertoire, then this could help the antibodies bind to and neutralize SARS-CoV-2, just as it was demonstrated in the aforementioned study for HIV-1, and in other classes of HIV-neutralizing human antibodies as well (Kanyavuz et al., 2019). For instance, by means of sulfation of antigen-binding sites of certain antibodies, the latter would be endowed with a strong negative charge, which would enable them to bind favorably to the unique—positively charged—inserts on the spike as identified in Sørensen et al. (2020).

Interestingly, a recent study identified that mechanisms other than those based on the traditional paradigm are at the core of coronavirus neutralization. Precisely, Wang C. et al. (2020), employing recombinant human mAb production, analyzed a human mAb that neutralizes SARS-CoV and SARS-CoV-2 using a trypsin-triggered cell-cell fusion assay. Remarkably, this study reveals that antibody binding to the virus does not compete with binding of the virus to the ACE2 receptor. This led to the conclusion that this particular mAb (47D11) neutralizes both “SARS-CoV and SARS-CoV-2 through a yet unknown mechanism that is different from receptor-binding interference.” The alternative mechanism for neutralization was not reported.

### 2.3. The Possible Role of More Unusual Mechanisms in the Multifarious Covid-19 Immune Response and Duration

It is important to realize that post translational antibody diversification mechanisms not only need to be expressed by the relevant cells, but that they are subject to selection during antigen-specific antibody responses (Van De Bovenkamp et al., 2018). It is conceivable that upon initial SARS-CoV-2 infection the immune system mounts a broad antibody response against certain viral components, and that this possibly exhibits cross-reactivities to other coronaviruses. A more refined and targeted response would only be effective when the necessary antibody diversification mechanisms are appropriately upregulated. Patients with a poor disease progression may lack these more specific mechanisms for virus neutralization in critical cells or tissue.

If post translational mechanisms (which are subject to adequate selection) play a significant role in immune response in Covid-19, then this may in part explain why patients recovered from Covid-19 quickly lose their antibodies (Long et al., 2020), why some do not have strong neutralizing antibodies to begin with, why infection may linger for some patients (Khamsi, 2020), why there is such a drastic diversity of neutralizing mAbs isolated from infected patients (Liu et al., 2020), or why there is such a marked diversity between neutralizing Ab titer and disease progression altogether (Jahanshahlu and Rezaei, 2020; Wu F. et al., 2020). If some cells or tissue do not harbor the appropriate post translational antibody modification machinery, or if it does not get adequately selected and upregulated, then this could result in a partial, incomplete or failed immune response, either locally or systemically. This could trigger additional rarely recognized antibody diversification mechanisms which then could escalate. In addition to targeting highly variable pathogens, alternative diversification strategies are also often used as a last resort response, with elevated “risk of unwanted reactivities, in particular, by overcoming immune- tolerance checkpoints” (Kanyavuz et al., 2019).

Indeed, a recent study (Dorward et al., 2020) found that death in Covid-19 is primarily an aberrant immune response, principally involving the lung and reticuloendothelial system, rather than pathogen mediated, organ inflammation and injury. This study revealed a variable but widespread distribution of viral RNA and protein, but a discordant inflammatory response to local viral presence, both between and within tissues.

The findings that viral components were identified in some cells and tissues without adjacent inflammation may indeed be due to the presence of antibody diversification mechanisms that have been sufficiently selected to neutralize the virus in some tissue or cell. On the other hand, their lack may explain the marked cell culture tropism of SARS-CoV-2 as recently confirmed by analyzing the SARS-CoV-2 infection and replication capacity in different cell lines and types. In particular, human liver cells and human embryonic kidney cells showed only modest viral replication. Significantly, human adenocarcinoma cells were incompatible with SARS-CoV-2 infection altogether (Harcourt et al., 2020).

### 2.4. A Response to Coronaviruses via Antibody Specific Conformational Dynamics

Another rarely recognized strategy for antibody diversification may be extremely relevant to coronaviruses - the role of conformational dynamics of the V region. Thereby, antibodies with highly pliable binding sites can bind many structurally unrelated antigens (reviewed in Kanyavuz et al., 2019). This may in fact be a predominant mechanism how our immune system deals with coronaviruses in general. As persistent and highly heterogenous pathogens, they are probably recognized by highly promiscuous antibodies. In fact, the recent findings of substantial T cell reactivity in patients that have never been exposed to SARS-CoV-2 most likely results from memory T cells derived from exposure to “common cold” coronaviruses (Sette and Crotty, 2020).

Thus, as pathogens with high antigenic variability such as coronaviruses employ various masquerading tricks (see also section 3.1 below), it seems plausible that their recognition and neutralization will require much more adaptable and refined immune response strategies than those afforded by gene sequence determined immunity alone.

Interestingly, the V regions of both heavy and light chains can be reconfigured by the incorporation of non-immunoglobin sequences in V genes. This is often realized by the insertion of an entire protein into the antigen binding site or by short nucleotide insertions and deletions (indels) (Kanyavuz et al., 2019). Notably, such changes in the sequence are often generated as a result of infections with highly mutable pathogens and can cause reconfiguration of the antigen-binding site, facilitating the accommodation of epitopes that are not easily accessible (Nicholls et al., 2003; Gu et al., 2005). Moreover, some of the integrations of non-immunoglobin proteins as part of their binding sites utilize alternative splicing options, either with or without these protein inserts. The practical significance of this is that through alternative splicing, this endows one antibody with plural antigen-binding specificities.

Thus, with continuously persistent variable pathogens the immune system often responds to different conformations and other more plastic and refined factors (Kanyavuz et al., 2019) rather than mere sequence defined epitopes. Conformational plasticity in broadly neutralizing antibodies may be critical to enhance antigenic specificity and neutralization capacity of distinct coronaviruses. The involvement of conformational dynamics as a key factor in adaptive immunity is known for distinct HIV-1 variants (Prigent et al., 2018) and was independently speculated in Wang C. et al. (2020) regarding the aforementioned unknown mechanisms of SARS-CoV-2 infectivity that are different from mere “receptor-binding interference.”

### 2.5. Autoimmune Responses in Covid-19

Conformational plasticity can lead to an enormous broadening of antigens that can be recognized. The flip side of such polyreactive antibodies is that they often cross-react with self-antigens as well, especially since promiscuous antibody mechanisms often get misdirected in the presence of co-morbidities and/or co-infections (Kanyavuz et al., 2019).

Autoantibodies have also been suggested as drivers of severe Covid-19 (see Khamsi, 2021, and references therein). Whether the autoantibodies identified in this context harbor modifications beyond the sequence-level or not has not been elucidated and would also require more detailed studies. As explained in Khamsi (2021), the autoantibody theory can explain several of the main mysteries involving Covid-19, such as, why some people get so much sicker than others, the involvement of additional organs (blood vessels, heart, and brain), and the varied response in disease clearance.

Details how this could happen are still unclear. Autoantibodies themselves could be spurred by Covid-19 (Zuniga et al., 2021). They are also a hallmark of more unusual antibody modification processes. The fact that the latter can be triggered by infection has been studied for mosquito-borne infectious diseases such as malaria. Apparently, malaria infection itself evokes a marked rearrangement of the genome of B cells; this genomic instability seems to be the molecular machinery to enable the incorporation of non-immunoglobin proteins into the V region of antibodies enabling them to recognize many unrelated antigens (Kanyavuz et al., 2019).

For SARS-CoV-2, the high similarity between numerous short sequences of the SARS-CoV-2 spike protein and human proteins (Kanduc and Shoenfeld, 2020; Sørensen et al., 2020) could lead to a multiplicative process, where the disease could further drive the body to produce more unusual antibodies, leading to a downward spiral. Therefore, just because the individual epitopes incorporated by, say a specific peptide vaccine do not have human homologies (as utilized in Sørensen et al., 2020), this does not mean that the entire antibody repertoire induced by such a vaccine, including the various unassessed modifications, will target these and nothing else.

### 2.6. Beyond Protein Interaction–the Use of Cofactors

Simply focusing on canonical antigen-antibody interactions is also missing immune response mechanisms involving non-canonical players. An interesting example is a human HIV-1 antibody called 2G12. Structural analysis of 2G12 revealed a unique architecture: a global rearrangement resulting in a dramatic structural reconfirmation of the antigen binding site (Calarese et al., 2003). Peculiarly, this particular binding site overlaps with binding sites of other HIV-1 antibodies. Yet, in contrast to these other antibodies, 2G12 can contact both protein and carbohydrate components of gp120 (Barnes et al., 2018).

By using cofactors, antibodies extend their functional activity far beyond that which would be possible with the mere use of amino acids from the polypeptide chain alone. The former range from metal ions to large organic compounds. Although such refinement strategies are believed to add a considerable selective advantage for pathogen neutralization, depending on the pathological state of the host, they may also increase the risk for a misguided immune response: those cofactors bound to these non-canonical antibodies may bind to other non-protein molecules of the host also.

A unique role of antibody specificity enhancement by the use of cofactor molecules is played by iron. Interestingly, in one study, exposure of pooled human IgG obtained from plasma of more than 3,000 healthy donors to Fe^2+^ ions lead to a considerable expansion of antibody repertoire diversity (Dimitrov et al., 2006).

The use of iron as a cofactor is essential to both pathogenic microorganisms and their host (Kanyavuz et al., 2019). It is suggested here that if misdirected, it may in part be responsible for the findings that death in Covid-19 is primarily an aberrant immune response. Notably, Dorward et al. (2020) revealed iron laden macrophages within bone marrow, despite the absence of typical causes of secondary iron overload. The authors raise the possibility that these macrophages may result from viral infection itself (and in turn play an anti-viral or tissue repair role) or that they are being activated as part of the wider immune response to the virus (and themselves promote vascular and tissue injury).

In the context of HIV and hepatitis C virus (HCV) infections, iron overload could partially be caused by the viruses themselves (Drakesmith and Prentice, 2008). Generally, in an oxygen-rich environment iron is highly insoluble and has low bioavailability despite its abundance. Therefore, as iron is essential for viral propagation, this is believed to lead to a “fierce competition between a host and its pathogens” (Drakesmith and Prentice, 2008).

Nonetheless, Figure 4 in Dorward et al. (2020) demonstrates a clear mismatch between consistent plasma cell abnormalities in spleen and mediastinal lymph node, and presence of the virus. Thus, it seems difficult to see why the observed elevated iron load could be caused exclusively by the virus itself. The lack of association between viral load and iron laden macrophages raises the possibility of a post translational immune response to be at least partially responsible for these plasma cell abnormalities. If the former involves the use of Fe^2+^ ions as a cofactor, then it is likely that the host will upregulate the entire iron metabolism machinery. This in itself could lead to oxidative stress, as demonstrated by a study investigating viral infection in fish with pancreatic necrosis virus. Therein, iron overload was clearly associated with oxidative stress induced by a massive upregulation of genes involved in iron metabolism (Tarifeño-Saldivia et al., 2018).

Overall, non-genetic immune response strategies are often evoked as a last resort to neutralize the virus, in part due to risk of unwanted reactivities (Kanyavuz et al., 2019). In the context of iron cofactors as a last resort strategy, the resulting iron overload could not only support the colonization of various pathogenic microorganisms (Drakesmith and Prentice, 2008), but also lead to oxidative stress, inflammation, and a decrease in levels of immunity (Drakesmith and Prentice, 2008).

In terms of Covid-19 pathology, Dorward et al. (2020) observed that “iron overload is an aberrant response deleterious to the host in Covid-19” consistent with the observation that with HIV and HCV infection, iron overload is associated with poor prognosis. Iron accumulation was identified as one of the key factors of fatal Covid-19 in Dorward et al. (2020), and better understanding its mechanism was highlighted as a top priority. It will be important to clarify if, and to what extent, this can be linked to beyond sequence determined immune responses pathways.

### 2.7. The Possible Role of Heme

Several studies have revealed a rather unique role of heme to enhance the overall tendency of antibodies to interact with proteins for which they are not initially specific. These novel binding specificities have been shown to be the result of heme directly binding to antibodies and then acting as an interfacial cofactor for engagement with the antigen (Dimitrov et al., 2007, 2014). It was revealed that this interaction of heme with antibodies leads to a significant diversification in reactivity. Significantly, this is not only toward pathogen-derived antigens but also toward autoantigens (Dimitrov et al., 2007; Gupta et al., 2015; Lecerf et al., 2015).

One may speculate that heme-sensitive antibodies could also play a significant role in the pathology of fatal Covid-19. While under homeostatic conditions heme is bound to various hemoproteins and sequestered intracellularly, it can be released extracellularly during pathological conditions, most notably oxidative stress (see section 2.6). It is well established that fatal Covid-19 most often occurs due to critical impairment of oxygenation, and one may speculate that this is further accentuated by misguided or overactivated adaptive immune system pathways.

Notably, under oxidative stress, some hemoproteins release their heme prosthetic groups. Non-protein bound heme is highly cytotoxic and free heme is very reactive. When heme is bound to antibodies, this not only leads to a substantial increase of their binding characteristics, but also to a drastic increase of their antigen-binding affinities (Hadzhieva et al., 2015). It is obvious that the unique physiochemical characteristics of “regulatory” heme (Kühl and Imhof, 2014) in terms of aromatic, hydrophobic, metal coordinating and anionic groups, leads to an enormous possibility for non-covalent interactions (Kanyavuz et al., 2019). The biological significance of this is unclear (Kanyavuz et al., 2019), especially related to auto-antigens and the presence of co-morbidities.

## 3. Implications for Vaccine Design

Non-genetic antibody repertoire diversification shows that the humoral immune response can produce antibodies that mimic a vast array of recognition and activation tactics far more complex than those defined by specific amino acid sequences alone. Nevertheless, non-genetic viral tricks can attenuate the efficacy of vaccines and complicate their development. For instance, the marked tissue tropism of viral antigen glycosylation is a well established problem with cell-culture based vaccine design. Previous studies involving the influenza virus have reported that the composition and relative abundance of glycans of some of its envelope glycoproteins markedly depends on the host cell lines used for virus production. This section analyzes related difficulties with Covid-19 vaccine design, as exemplified on Biovacc-19 (section 4.3).

### 3.1. The Difference Between Recognizing and Neutralizing Antibodies May in Large Part Depend on Frequently Neglected Antibody Diversification Mechanisms

As viruses keep evolving, one of the key challenges in adaptive immunity is that many antibodies are only able to bind to certain viral components. They are not neutralizing in that they do not bind the virus in a manner that blocks infection. However, several beyond sequence determined strategies to broaden the antibody repertoire are sufficient to achieve neutralization.

For instance, just as glycans are a key component of all coronaviruses to achieve immunoevasion, they are also a key component of an enhanced adaptive immune response. Notably, a recent study (Walls et al., 2019) identified some unexpected masking functions of SARS-CoV and MERS-CoV S glycoproteins, and how specific antibody diversification strategies are able to mirror and block these masquerading tricks.

In general, the spike (S) glycoprotein in coronaviruses has long been recognized as critical for entry into host cells. S is composed of two functional subunits that are responsible for binding to the host cell receptor and fusion of the viral and cellular membranes, respectively. It has long been believed that receptor binding automatically initiates membrane fusion. Interestingly, however, its receptor binding domain (designated domain B) exhibits multiple conformational states that modulate the accessibility of the receptor-binding motifs (RBMs) and thereby the ability to interact with host cells. As pointed out in Walls et al. (2019), the pre-fusion spike of various coronaviruses adopts a conformation of domain B which is incompatible with receptor engagement. This closed conformation is only a masquerading trick. Via specific structural rearrangements, the spike acquires an open configuration, and this then makes membrane fusion possible.

What is even more striking is that specific anti-SARS-CoV antibodies mirror this uncommon membrane-fusion activation pathway just described. That is, on the basis of non-genetic antibody diversification, some rare antibodies achieve their potent neutralizing activity by mimicking this exact same process of receptor attachment and conformational rearrangement. This way, the masquerading strategy of the virus is mirrored and thereby effectively targeted.

Although Walls et al. (2019) showed that the antibodies to both SARS-CoV and MERS-CoV block attachment to the host cell receptor, only the anti-SARS-CoV S antibody triggers fusogenic conformational changes via receptor functional mimicry.

Clearly, viruses rely on, and hence utilize, the host protein synthesis machinery for their own purposes. Not surprisingly then, many coronavirus proteins are modified by various kinds of co- and post-translational modifications. By introducing new functional groups such as phosphates or carbohydrates, or by alternative cleavage strategies, this vastly extends the repertoire of the 20 standard amino acids. These modifications are important for folding, structure, stability, and intracellular sorting of proteins. They have effects on receptor binding, fusion activity, and antigenic properties of the virus (Oostra et al., 2006; Fung and Liu, 2018).

Some of the key difference between SARS-CoV and SARS-CoV-2 may be rooted in co- or post-translational modifications. In particular, Andersen et al. (2020) highlight the role of O-linked glycans in related coronaviruses. In fact, already pre-Covid-19, glycans have been recognized as a critical factor to enable zoonosis (Yang et al., 2015), and various mutants of the SARS-CoV 3a and M protein helped to elucidate residues with high O-glycosylation propensity scores already in 2006 (Oostra et al., 2006).

Modifications such as the shielding by glycans and various reconfigurations make epitopes on the spike inaccessible to usually considered antibodies and may be key factors to evade a neutralizing immune response. Correspondingly then, it seems reasonable to conclude that additional antibody mechanisms other than genetic are required to provide neutralization (Figure 1), and that targeting SARS-CoV-2 via traditional antibody responses may indeed miss the key fact of how this novel virus operates.

### 3.2. The Vaccination Response Is Very Much Dependent on the Vaccinee

**Figure 4:**
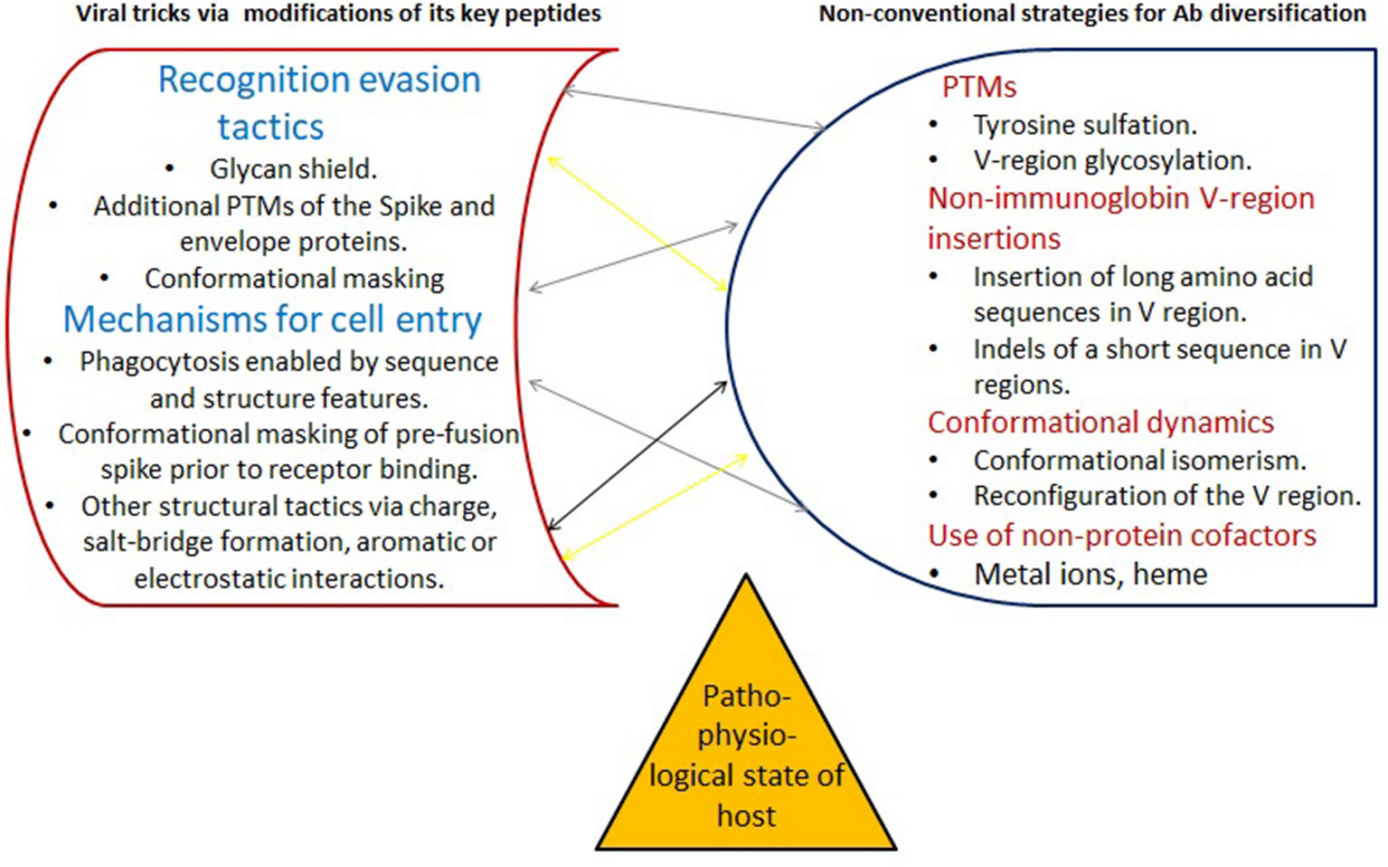
Non-genetic modification strategies on both sides. Right: Adaptive immunity relies on sophisticated genetic mechanisms to create the diversity of antibody V regions. Several rarely recognized routes for diversification of antibody specificities (left) add an increased level of adaptability and plasticity, far beyond that encoded in the genome or generated by acquisition of somatic mutations [reviewed in Kanyavuz et al. (2019)]. They may be necessary to combat the various tricks viruses are using which are also far beyond what is encoded in their linear amino acid sequence (right). Both, the immune system and the virus, likely evoke various strategies simultaneously. Depending on their context (e.g., the pathophysiological context of the host, interrelationship with the other arms of the immune system - not depicted), some of the beyond sequence determined diversification mechanisms may not be available or adequately expressed. Left: PTMs also apply to viral proteins. They add temporal and spatial regulation to modulate their functions, in particular to enhance viral immune evasion tactics. PMS, post-translational modifications - the covalent modifications of polypeptides after they are synthesized. Ab, antibody.

As noted, beyond sequence dependent strategies of antibody diversification are frequently elicited in response to infections with highly mutable pathogens (Kanyavuz et al., 2019). In such a situation, a fixed set of epitopes (as utilized for prophylactic treatment) may not trigger a sufficient neutralization immune response. This seems to be supported by the observation that these refined strategies are not governed by sequence dependent determinants alone (Figure 4).

In healthy humans, 15–25% of IgG antibodies have N- linked glycan structures in their V regions (van de Bovenkamp et al., 2016). Importantly, however, specific patterns of Fab glycosylation are concurrent with physiological and pathological conditions (van de Bovenkamp et al., 2016). The frequency of antibodies modified this way can increase considerably in some conditions such as rheumatoid arthritis and Sjörgen syndrome (see Kanyavuz et al., 2019 and references therein). In naive B cells, the germline sequences are almost devoid of glycosylation modifications. On the other hand, it has been found that mutations leading to glycosylation in the V regions are the consequence of a somatic hypermutation process (Hamza et al., 2015; van de Bovenkamp et al., 2016; Van De Bovenkamp et al., 2018). Many of these antibody diversifications are implicated with disease conditions. For example, 80–100% of autoantibodies recognizing citrullinated proteins in patients with rheumatoid arthritis contain Fab-bound glycans.

Overall, antibody composition and function is not dictated by a fixed choice of epitopes alone, such as e.g., suggested by Sørensen et al. (2020), but will be heavily influenced by genetics, environmental context, and the general physiopathological state of the vaccinee, including their preexisting infection and immunization status relative to coinfections.

### 3.3. The Infeasibility of Predicting and Modeling the Antibody Response From the Epitope Sequence Alone

In contrast to more traditional vaccines, synthetic vaccines utilize short amino acid sequences of the immunogenic protein (epitopes) to evoke the anticipated antibody response. As this approach distinctively relies on the specific amino acid sequence of the immunogenic protein of interest, this is believed to induce a direct and potent immune response. Furthermore, the same approach is deemed sufficient to target viral mutations, by incorporating additional epitopes into the vaccine design. Nonetheless, to effectively combat SARS-CoV-2 evasion strategies, information on the residue level alone is likely going to be insufficient.

Limitations of approaches that only target specific epitopes, as well as the role of glycans in the evolution of this virus, was recently demonstrated by Andreano et al. (2020). The goal of this study was to identify whether the virus, under the selective pressure of the polyclonal immune response in convalescent or vaccinated people can evolve to escape herd immunity and antibody treatment. By co-incubating authentic virus with a highly neutralizing plasma from a Covid-19 convalescent patient, it was found that while initially the plasma fully neutralized the virus, after several passages the virus exhibited numerous mutations which generated a variant completely resistant to plasma neutralization. Significantly, the final modification which completely eliminated antibody neutralization was attributed to the introduction of a new N-glycan.

### 3.4. The Infeasibility of Predicting the Full Spectrum of the Adaptive Immune Response

Fully predicting the complete antibody repertoire triggered by all the non-genetic modifications (both, at the viral and the immune response side, Figure 4) seems like a daunting task. Therefore, it is very challenging to assess their full range of action. In the context of a peptide vaccine, these unknown modifications may trigger unanticipated recognition and binding pathways. For example, in the case of infection of blood cells with *Plasmodium falciparum*, rarely recognized antibody diversification involves a large protein insertion which encodes an inhibitory receptor (LAIR1) that normally binds to collagen (Kanyavuz et al., 2019). Importantly, this foreign segment carries various somatic mutations that ablate the binding to collagen but confer increased affinity for *P. falciparum* antigens. Surprisingly, these antibodies directly use LAIR1 protein for the recognition of malarial antigens. A concern with this is that the reverse type of cross-linking is possible also: non-genetic modifications may mediate increased polyreactivity and binding affinity of certain antibodies. As noted above, increased reactivity toward autoantigens is one of the most critical concerns with beyond sequence determined modifications, especially since their frequency and selection is very much dependent on the physiopathological state of the host.

### 3.5. More Refined Mechanisms Influencing ADE

Safety concerns about coronavirus vaccines were first raised years ago, given the observation of antibody-dependent enhancement (ADE) both *in vitro* and in animal studies. In particular, Wang et al. (2016) identified SARS-CoV B-cell peptides that exhibit disparate functions in protection and enhancement against SARS-CoV *in vitro* and in experimental monkeys. They revealed that a specific epitope which was immunodominant and reactive with its cognate neutralizing Ab, was nonprotective and even harmful when used as a vaccine. In Wang et al. (2016), the aim was to elucidate the mechanism and identify the sequence responsible for this disparate functionality. It is suggested that this ADE enabling mechanism distinctively mirrors certain rarely recognized processes involved in the generation of antibody diversification. This section further analyzes these findings.

**Figure 5:**
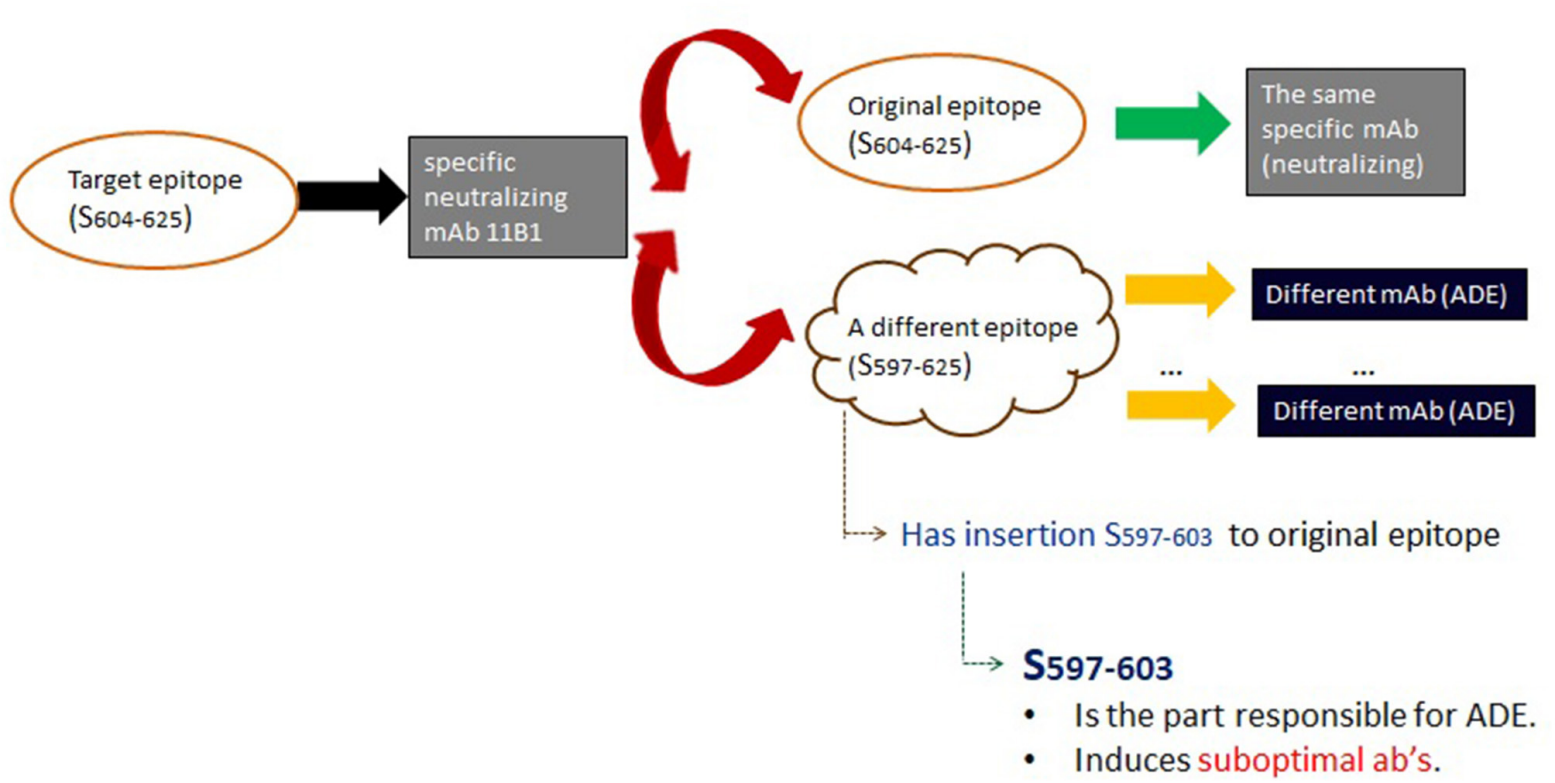
Analysis of the mechanism in Wang et al. (2016) of ADE in SARS-CoV. Wang et al. (2016) demonstrated that an antibody targeting a specific epitope of the SARS-CoV spike protein can enhance virus infection both *in vitro* and in the experimental monkeys. Significantly, this was linked to a clear epitope sequence-dependent mechanism [see Table 1 in Wang et al. (2016) for nomenclature]. While the targeted epitope induced (black arrow) the anticipated and desired antibodies (gray box), the latter cross-reacted (red arrows) with two epitopes. The first was the original epitope (top), resulting in the expected neutralizing antibodies. However, the second epitope (bottom) led to ADE antibodies (black boxes). The critical feature elucidated by Wang et al. (2016) is that the second epitope (bottom) is merely the original epitope, with an additional N-terminal sequence insert (*S*_597−603_). This insert, then, because it is itself immunogenic, was found to be responsible for inducing the enhancing function. However, it only induces sub-optimal antibodies. It is possible that binding of these enables changes in the spike protein, or otherwise catalyzes SARS-CoV attachment to and/or membrane fusion with target cells. The *in vitro* experiments in Wang et al. (2016) that demonstrate this in Vero E6 cells are particularly striking as the latter lack *FcγR*, and the best understood mechanism for ADE involves *Fc*-mediated internalization and replication of virus.

In Wang et al. (2016), the factors enabling the disparate functions were identified as follows (Figure 5). At first, epitopes were assembled into immunogenic peptides. Consequently, it was shown that the reversing of the functionality of the induced antibodies were fostered by rather small modifications of one of those peptides. Using the nomenclature of Table 1 in Wang et al. (2016), Abs against peptide sequence *S*_604−625_ were shown to have a neutralizing function; on the other hand, two Abs against sequence *S*_597−625_ have the ability to enhance infection. Surprisingly, however, the difference between these two sequences is that the latter is merely the former with an additional 6 amino acid insert. These findings show that even if one epitope evokes clear neutralizing antibodies, a very simple modification thereof (e.g., an insertion) may reverse its function.

The differentiation between enhancement or protection hinges upon a simple amino acid insertion in an epitope recognized by an Ab which by itself has a neutralizing function. This insertion triggers Ab's with the opposing function, possibly by exposing specific conformations that catalyze SARS-CoV attachment to and/or membrane fusion with target cells. This highlights the role of conformation beyond that of traditional antigen-antibody interactions.

To reiterate, at the core of such disparate function is, firstly, dual (or plural) specificity of specific antibodies; secondly, in Wang et al. (2016) this was linked to the difference between *S*_597−625_ and *S*_604−625_ via the sequence insert. It seems plausible that analogously to an insertion, a similar situation could unfold via epitope modifications that happen beyond the sequence level, and/or non-genetic antibody diversification strategies that enable them to bind to different epitopes. If, for instance, these modifications involve the addition of some sugars or other cofactors, then this could significantly impact the conformation in some viral entry protein that accelerate membrane fusion and thereby could make a viral infection even worse.

Wang et al. (2016) rely on an elaborate analysis to identify all the epitopes that could evoke ADE. This was made possible by chemically ligating together different candidate sequences to prepare a multiple antigen peptide system. This system was used to vaccinate four rhesus monkeys via a suitable inclusion-exclusion design relative to the critical epitopes in question. This way, it was possible to clinically distinguish protective peptides from those that induce enhancement. With 33 epitopes (as in Biovacc-19), any and all combinations enabling disparate functions will become a rather significant challenge and, as in Wang et al. (2016), would still require extensive clinical experiments to rule out ADE.

Thus, apart from performing rigorous experiments, it is unclear how the function of the various peptides can be determined, in particular in light of co- or posttranslational modifications. Separate amino-acid sequences alone are a poor predictor of this function.

For SARS-CoV-2, the issue of ADE, both for disease enhancement and as implied in inflammatory responses, remains hotly debated (see e.g., Lee et al., 2020). It is also not clear if ADE pathways are selected and upregulated in the context of highly variable pathogens. Given that the latter often trigger various alternative strategies to enhance antibody diversity and response, this seems plausible. Significantly, polyreactivity and autoreactivity are significant known risks associated with post translational antibody diversification mechanisms. Although not analyzed in the context of ADE, it is tempting to speculate that diversification strategies that happen beyond the sequence level (e.g., via the involvememnt of glycans) could be additional important factors in ADE.

### 3.6. Threshold Conditions in ADE

In addition to their involvement in ADE as postulated above, beyond sequence determined strategies may also play an indirect role, via their flexibility to influence the neutralizing potency of an antibody, as follows.

Most studies have focused on the Fc-gamma receptor in the involvement of ADE. Yet, additional mechanisms related to viral uptake and virus replication pathways are known also (Wang et al., 2016). For instance, in the case of West Nile Virus, it was found that there is an antibody occupancy threshold on the virion to either lead to neutralization or enhancement of virus infection (Pierson et al., 2006; Wang et al., 2016). That is, weakly neutralizing antibodies require a much higher Ab occupancy for neutralization, and significantly, when their occupancy falls below the threshold for neutralization, ADE can occur (see also Figure 5).

This raises the question if something similar could happen in cells that rely on post translational antibody diversification strategies for virus neutralization. Significantly, recent studies involving patients recovered from Covid-19 show that the majority of the induced antibodies are weak (Robbiani et al., 2020). Among other reasons, this may be due to a lack of an appropriate post translational immune system response and/or due to the fact that this study was not designed to identify such rarely recognized types of antibodies.

It is important to realize that here those weak antibodies were triggered upon natural infection. On the other hand, if prophylactic treatment does not evoke antibodies with stronger neutralization potential than described above, then the adaptive immune system may try to cope in several ways. Firstly, it could try to upregulate various strategies to enhance antibody binding and neutralization capacities (if supported in those cells). Unfortunately, such a response cannot be primed, targeted, or upregulated via vaccination approaches that work on the basis of sequence dependent interactions only (Figure 4). Another way the immune system may respond then, is to try to increase the level of the weak antibodies. However, if it fails to raise this beyond the required threshold, this may, analogously to what is observed with West Nile Virus (Pierson et al., 2006), actually elicit an ADE response.

## 4. Discussion

### 4.1. Challenges With Identifying More Unusual Antibody Diversification Mechanisms

This contribution hopes to draw attention to a much larger repertoire of adaptive immune response mechanisms induced with Covid-19, and to highlight the possibly significant role of these largely un-assessed strategies. It is intriguing why these have failed to receive broader attention. This may be due to several reasons:

- Most potent neutralizing antibodies are often only produced by very rare B cells (Tan et al., 2016; Walls et al., 2019; Robbiani et al., 2020). That is, not all B-cells are able to express various antibody diversification processes, and even if they do, the latter have to be positively selected. The marked plasticity of these mechanisms, including their spatial and temporal variations among various population groups, pose a significant challenge to their elucidation in a laboratory setting.
- The functional activity of such antibodies often requires the presence of specific agents. For example, the appearance of novel antibody reactivity against different phospholipids was only possible when whole human blood or plasma was incubated with heme-containing medium (McIntyre, 2004).
- Being able to predict which of the peptides in a SARS-CoV-2 protein are antigenic, and utilizing exclusively these for vaccine design, has the advantage that numerous pseudotyped viruses can be designed and analyzed outside of high security (BSL-3 or 4) environments. Nonetheless, the imperative to quickly build novel platforms and systems has prompted a narrow focus on specific antibody-antigen interactions; these incorporate only selected factors such as SARS-CoV-2 spike (S) protein and ACE-2 receptor involvement (e.g., Weisblum et al., 2020; Wu F. et al., 2020; Wu Y. et al., 2020). But unfortunately, these platforms are not equipped to analyze more unusual mechanisms or interactions. They not only miss advanced viral immune evasion strategies such as via co- or post-translational modifications (Oostra et al., 2006; Fung and Liu, 2018), but also their immune response counterparts (Figure 4).

Identifying beyond sequence dependent immune responses is not an easy task, particularly also since critical concerns regarding the use, testing, and authentication of usually considered antibodies have been raised. Nonetheless, the past few years have seen considerable improvements to help confirm their integrity, specificity and selectivity (Voskuil et al., 2020). Although the elucidation of more unusual antibody diversification strategies will require additional efforts, advances in techniques such as mass spectrometry, cryo-EM, and next-generation sequencing techniques have already proven extremely useful in this regard (Van De Bovenkamp et al., 2018; Walls et al., 2019).

### 4.2. Pointers to the Involvement of Such Mechanisms

The immune response in Covid-19 is manifold, very complex (Azkur et al., 2020; Vardhana and Wolchok, 2020), and is highly influenced by factors such as the pathophysiological state of the host, genetics, and even geographic location. Some of the clinical presentations of patients with severe Covid-19 have been attributed to a dysregulated innate immune response (Vardhana and Wolchok, 2020). Yet, innate immune hyperactivation is likely not the sole pathological driver in severe SARS-CoV-2 infections. While many arms of the innate immune response are potentially activated by SARS-CoV-2, the clinical toxicity observed during Covid-19 infection may in large part be a product of adaptive immune dysregulation (Vardhana and Wolchok, 2020).

Severe or fatal Covid-19 may not be consequent to failure of the more traditional adaptive immune system, but the manifestation of a much more involved misguided immune response (Dorward et al., 2020; Vardhana and Wolchok, 2020). Already pre-Covid19, studies with patients recovered from various coronavirus infections suggested that the adaptive immune system response involves mechanisms beyond those defined in terms of amino acid composition (Yang et al., 2015; Walls et al., 2019). The widely varied response to SARS-CoV-2 infection may be associated with frequently neglected strategies for remodeling variable (V) regions, due to their potential to add an enormous layer of plasticity, readiness, and adaptability. In relation to Covid-19, several recent findings point to their involvement:

- The discovery of the human monoclonal antibody 47D11 which neutralizes SARS-CoV and SARS-CoV-2 through a “yet unknown mechanism that is different from receptor-binding interference” (Wang C. et al., 2020).
- Widely varying neutralizing titers relative to neutralization activity. One of the first studies to test antibody neutralization against SARS-CoV-2 pseudovirus showed widely varying numbers (Liu et al., 2020). Paradoxically, the patients chosen for Ab isolation were those with highest plasma virus-neutralizing titers, but they were also those that presented with severe disease. The absence of a clear association between neutralizing Ab titer and disease progression was also observed by others (Jahanshahlu and Rezaei, 2020; Wu F. et al., 2020). This may be due to the fact that traditional antigen-antibody responses may be insufficient to achieve humoral immune naturalization.
- A marked diversity of neutralizing antibody response within and between individuals (Weisblum et al., 2020). Each of the four convalescent plasma samples tested in this study had distinct neutralizing activities. Most notably, it was found that the overall neutralization activity of plasma is beyond that afforded by the action of the specific antibodies alone. In essence, their findings suggest a much more refined, individual and plastic immune response processes than via sequence based epitope specific antibody mechanisms alone.
- Lack of association between systemic viral load and disease progression. Immunocompromised and severely ill patients generally tend to have a higher viral load (Jacot et al., 2020). These patients are also those for whom refined antibody diversification strategies tend to go awry, even systemically (Sect 3.2). On the other hand, fatal Covid-19 disease seems to be related to an inadequate or misguided immune response in specific tissue or cells rather than systemically (Dorward et al., 2020). This observation points to more refined processes and is also in line with a recent study that monitored viral loads over time and across several different patient populations (Jacot et al., 2020). When analyzing RT-PCR results according to gender, age, and health units, no significant differences were detected. This includes comparisons between pediatric age group and adults, and patients in the ICU and other hospital units. Overall, it was found that there is not direct link between viral burden and disease progression or even mortality. By contrast, recent experiments demonstrated huge variations in the capacity of SARS-CoV-2 to infect and replicate in different cell lines (Harcourt et al., 2020). Again, taken together, these findings suggest that the most critical factors of the learned immune response may not be its ability to reduce viral load at the systemic level, but about the presence or absence of more refined mechanisms in individual tissue and cells, as dictated by the host's pathophysiologic state.

### 4.3. Biovacc-19 and Beyond

If vaccine design is solely based on adaptive immune responses defined by specified amino acid sequences, this misses critical issues, as exemplified here in the case of Biovacc-19 (see Table 2 for details). Notably, in Sørensen et al. (2020) it is claimed that for this vaccine the possibility of inducing AI response or ADE is minimal. Unfortunately, based on the arguments reviewed and developed in this study, this is not the case. Likewise, the claim that Biovacc-19 covers all the various cell receptor binding options, and therefore denies virus binding, is unsubstantiated. Analogs concerns about safety and efficacy apply to other vaccines which exclusively target adaptive immune responses defined in terms of amino acid composition.

**Table 2:** Analysis of the claims made in Sørensen et al. (2020), regarding safety and efficacy of Biovacc-19.

**Design principle to fulfill key goal**	**Goal to be targeted**	**Comment**
Unique mechanisms for cell entry	“[T]he Covid-19 pandemic is revealing neurological, haematological and immunological pathogenicity in the virus which cannot be explained by the ACE2 receptor alone.” “that SARS-CoV-2 can enter cells without using the ACE2 but also by promiscuous attachment has implications for. vaccine development strategies.”	Unfortunately, fully predicting promiscuous attachments and recognition via the amino acid sequence alone is not sufficient (see section 3 and Figure 4).
	“These data. confirm that accumulated charge from inserts and salt bridges are in surface positions capable of binding with cell membrane components other than the ACE2 receptor.”	These results actually show that the virus uses mechanisms and interventions in addition to ACE2 receptor binding. These known facts are critical for vaccine design development.
Viral recognition evasion tactics	“We. discover what is - and is not - revealed at amino-acid level analysis of sequences by previous authors and to elicit the general mode of action for infectivity of this virus.” “In order to ensure that Biovacc-19 covers all the various cell receptor binding options, combined with our guiding criterion of using non-human like (NHL) epitopes, we systematically blasted (Uniprot P0DTC2) the Spike protein.”	Although fundamental levels of the adaptive immune response rely on the amino acid sequence of immunogenic proteins, this is not exhaustive as the immune system is able to use more refined recognition and response strategies (see text).
Safety considerations to minimize ADE	Regarding the *a priori* use of a specific adjuvant to enable TH-2 response to the peptide specific epitopes: “The benefit of using this strategy compared to conventional virus, Ribonucleic acid (RNA) or other vector-based vaccine systems is that the immune system will be guided *directly* to the epitopes which are relevant for virus neutralization.” Antibody-dependent enhancement (ADE) is low “[s]ince the antibodies are directed toward the receptor binding domain and other co-receptor domains.”	•This does not mean that the immune system may be responding to other factors, beyond the criterion involving the fixed 6 amino acid sequences, such as conformational dynamics, charge, non-protein cofactors, etc. •The rationale in Sørensen et al. (2020) also does not consider the potential of bystander activation. This is a known key mechanism (Vadalà et al., 2017) to provoke ADE whereby microbial agents (and this could include vaccines) release sequestered self-antigens from host tissue, triggering the activation of antigen-presenting cells dormant autoreactive T-helper cells, resulting in an additive effect of inflammation, cytokine release, and the recruitment of additional T-helper cells. •Some of the known ADE mechanisms are highly reminiscent of those employed by more unusual antibody diversification strategies, but have previously not been assessed in this light (see sections 3.5 and 3.6). Antibodies with pliable antigen-binding sites and conformational heterogeneity are capable of binding to many structurally unrelated proteins. More generally, the effect of more unusual strategies for remodeling variable (V) regions on ADE are unclear.
Safety considerations to minimize AI	“Biovacc-19's Method of Operation is upon non human-like (NHL) epitopes in.SARS-CoV-2's Spike protein.” “Non Human-Like (NHL) sequences found in SARS-CoV-2 spike protein S1.were obtained by blasting the Spike protein sequence using moving window of 6 amino acids in steps of 1 against the human protein sequence database.”	As mentioned in Sørensen et al. (2020), antibodies can only recognize 5-6 amino acids. Based on this, it is not clear why the analysis was not complemented by a 5 amino acid rolling window search. Also, as mentioned in Sørensen et al. (2020), other binding options are possible that use as little as four amino acids.
	“A further advantage of using NHL epitopes is that the immune system is free to mount robust, broad and long-lasting immune responses without being limited by local or even systemic immune-toxic reactions against our own human protein epitopes.”	The conclusion is unsubstantiated as we do not know what exactly the immune response will look like in each individual, and in susceptible tissues and cells. The full range of effects that could be triggered by vaccination or infection following vaccination is not known, as beyond sequence determined strategies may ablate binding to anticipated antigens and/or confer increased affinity for other proteins. It is unclear what that means in terms of (auto)polyreactivity based on the specific pathophysiological state of the host, and in relation to the other arms of the immune system.

In spite of the limitations, Sørensen et al. point to novel features of Covid-19 vaccine design that are compelling: first, the need to better understand viral strategies beyond mere sequence dependent mechanisms (section 2). In particular, coronavirus S glycoproteins are decorated with an extensive glycan shield which is known to support viral evolution, immune evasion, and which may also extend their host range. Second, Sørensen et al. (2020) stress the importance for a combined and overarching approach, to integrate the viruses etiology in disease pathogenicity with therapeutic considerations and vaccine design.

With Covid-19, more and more studies are pointing to the unique role of the host (Cheng et al., 2020; Dorward et al., 2020). It is therefore of paramount importance to consider a broader mechanistic framework - both, related to natural immune response processes but also for vaccine R&D). If it is really the case that SARS-CoV-2 exhibits, in addition to ACE2 receptor binding, a non-receptor dependent phagocytic method of action that not only explains its mechanisms of infectivity but also clinical evidence of pathogenicity, then this means that these paradigms are intimately connected. If so, then questions related to infectivity and immunity cannot be separated from questions related to morbidity, and the overall state of the host.

## 5. Conclusion

SARS-CoV-2's etiology involves various sophisticated mechanisms, as is highlighted by the developers of Biovacc-19 (Sørensen et al., 2020), and suggested elsewhere (Andersen et al., 2020; Cheng et al., 2020). The glycan shield of coronavirus spikes in general, and specially linked oligosaccharides of this new virus in particular, are major determinant of host range and tissue tropism. Years ago, it was already established that the glycosylation of surface antigens of specific viruses alters the ability of the host to raise an effective immune response and also attenuates the efficacy of existing vaccines (Hütter et al., 2013). However, little is known about the various immune response strategies to counter viral evasion strategies during SARS-CoV-2 infection.

The involvement of these strategies may often remain undetected, not only because they have not received much attention. Although in very specific cases they were associated with most potent antibodies already years ago, their elucidation has proven elusive, and their emergence, function, and regulation are still poorly understood.

New generation vaccine design strategies try to predict which of the peptides in a pathogenic protein are likely to be antigens. However, peptides modified via additional processes such as glycosylation, conformational plasticity, charge, or steric hindrances offer more sophisticated options for viral immune evasion than can be predicted by sequence based epitopes alone. In a successful immune response, the advanced masquerading mechanisms employed by SARS-CoV-2 are likely mirrored by alternative immune response strategies. Notably, however, these can overrule usually considered adaptive immune responses in both natural infection and vaccination.

Here, Biovacc-19 is used as a case study. Nonetheless, the findings apply to other vaccines whose design principle is rooted in an epitope sequence dependent approach and more traditional adaptive immune response strategies to thwart various binding options of the virus. Relying on these alone can threaten the prospect of safe and effective vaccination platforms designed without these considerations.

Since the first submission of this article, several others have articulated the need to better comprehend the role of glycans during SARS-CoV-2 infection (Table 3). At the same time, Covid-19 vaccines have been developed and rolled out globally in an unprecedented effort. Ongoing mutations of the virus, continued breakthrough infections (Centers for Disease Control and Prevention, 2021), and detailed laboratory experiments point to the prospect of reinfection with antigenically distinct variants and raises concern about reduced efficacy of spike-based vaccines altogether (Robbiani et al., 2020). In addition, the concern arises that SARS-CoV-2 could increasingly employ non-genetic immunoevasion strategies which are not specifically targeted by sequence based vaccine platforms and which may make it resistant to a wide range of prophylactic and therapeutic treatments.

**Table 3:** The importance of glycans in coronaviruses with special focus on SARS-CoV-2.

	**Key features beyond nucleotide-based determinants**	**Source**
More refined features of epitopes	The N-glycans on S protein play important roles in proper protein folding, stability, dynamics, and priming by host proteases.	Lorenzo et al., 2020; Walls et al., 2020; Watanabe et al., 2020; Zhang et al., 2020
	Glycosylation shapes viral tropism. Glycan modification can be heavily influenced by the producer cell, resp. the cellular expression system used.	Goh and Ng, 2018; Watanabe et al., 2020
	Glycosylation of proteins is a PTM and is processed by various enzymes coordinated in the endoplasmic reticulum and Golgi apparatus.	Watanabe et al., 2020 and references therein.
Relevance to Covid-19 immune response.	Glycosylation is a key structural feature of the S protein that eludes detailed experimental structural characterization.	Lorenzo et al., 2020
	It is unknown whether convalescent sera of SARS-CoV-2 individuals recognize the “glycan cloud.”	Wintjens et al., 2020
	Glycosylation sites are under selective pressure to facilitate immune evasion by shielding of specific epitopes.	Watanabe et al., 2020
	Glycans obstruct receptor binding and proteolytic processing during antigen presentation and alter the accessible surface areas on the S-protein, impeding antibody-antigen recognition.	Wintjens et al., 2020; Zhang et al., 2020
	The glycan masking of epitopes may address why some patients' sera is limited in its ability to prevent virus entry into host cells.	Walls et al., 2020; Wintjens et al., 2020
	Viral mutants may evade host antibodies without changing the epitope sequence, but rather by changing the glycan chain orientation.	Wintjens et al., 2020
	The extensive tissue tropism of glycosylation may affect glycan structure and dynamics and thereby expose cryptic epitopes.	Lorenzo et al., 2020
Findings of glycoproteomics studies.	The SARS-CoV-2 trimeric spike is highly glycosylated. Both N- and O-glycosylation of the spike likely play a role in protein folding and immune evasion.	Shajahan et al., 2020; Watanabe et al., 2020; Zhang et al., 2020
	Receptor bindings sites on the SARS-CoV-2 spike are shielded by proximal glycosylation sites (esp. when the RBD is in the ‘down' conformation).	Watanabe et al., 2020
	Glycan modifications are present at a critical binding location of the spike protein.	Shajahan et al., 2020
	N-glycosylation may be associated with the recognition of RBD to the ACE2 receptor.	Zhang et al., 2020
	S protein expressed in different cells display distinct N-glycosylation patterns.	ibid.
	The glycosylation of the S protein in human cells exhibits remarkable heterogeneity on N-glycosites, with the N-glycan types primarily determined by the host cells regardless of the location of the glycosites.	ibid.

Surprisingly, although still poorly understood, even much of the effectiveness of mRNA vaccines may rely on mechanisms more complex than dictated by specific amino acid sequences alone. Indeed, the CureVac mRNA vaccine candidate recently led to considerable confusion as preliminary data from a 40,000 person trial showed an about 50% reduced efficacy compared to the mRNA vaccines by Pfizer/BioNTech and Moderna. Although viral variants and disparate statistics could have contributed to such a stark difference, some believe that the chemistry of the vaccines has much to do with this. Both of the mRNA vaccines by Pfizer/BioNTech and Moderna employ modified nucleotides while CureVac uses unmodified RNA (Dolgin, 2021). This modification was originally invented to prevent over-reactive immune responses to mRNA vaccines (Pardi et al., 2018). By their very design—again, involving only genetic information of the spike—such added influences beyond the naked mRNA sequence, or those fostered by the different carrier systems, cannot appropriately mimic any co- or post translational evasion tricks of the virus. This leads to the question to what extent more traditional vaccines implicitly address such beyond sequence dependent factors.

As SARS-CoV-2 continues to be an ongoing burden to the entire world, there is a new urgency to tackle previously neglected questions in human immunology. To move the field forward and optimize our comprehension of this viruses interactions with its host, we must explore influences and factors beyond mere sequence level determinants.

## Data Availability Statement

The original contributions presented in the study are included in the article, further inquiries can be directed to the corresponding author.

## Author Contributions

The author confirms being the sole contributor of this work and has approved it for publication.

## Conflict of Interest

The author declares that the research was conducted in the absence of any commercial or financial relationships that could be construed as a potential conflict of interest.

## Publisher's Note

All claims expressed in this article are solely those of the authors and do not necessarily represent those of their affiliated organizations, or those of the publisher, the editors and the reviewers. Any product that may be evaluated in this article, or claim that may be made by its manufacturer, is not guaranteed or endorsed by the publisher.
